# Navigating barriers and enablers to academic success for students with disabilities at the University of Gondar, Ethiopia: A participatory action research approach

**DOI:** 10.4102/ajod.v15i0.1975

**Published:** 2026-08-07

**Authors:** Molalign B. Adugna, Tadesse A. Tedla, Mustofa W. Jemal, Getachew G. Tadese, Bilen M. Araya, Missaye M. Mengistie, Biset Y. Abera, Tibebu Guadie, Roza A. Abate, Yitayal A. Mengistu, Hiwot A. Mekuanint, Selamawit Kassa, Angela Coderre-Ball, Madison Robertson, Rylan Egan

**Affiliations:** 1 Department of Sociology, University of Gondar, Gondar, Ethiopia; 2 Department of Special Needs, Faculty of Education, University of Gondar, Gondar, Ethiopia; 3 Department of Communication and Journalism, University of Gondar, Gondar, Ethiopia; 4 Department of Social Work, University of Gondar, Gondar, Ethiopia; 5 Department of Rehabilitation Science, Queen’s University, Kingston, Canada; 6 Department of Psychology, University of Gondar, Gondar, Ethiopia; 7 Department of Law, University of Gondar, Gondar, Ethiopia; 8 Department of Special Needs, University of Gondar, Gondar, Ethiopia; 9 Department of Law, Queen’s University, Kingston, Canada; 10 School of Nursing, Health Quality Programs, Queen’s University, Kingston, Canada

**Keywords:** barriers, disabilities, education, Ethiopia, participatory action research, stigma, enablers

## Abstract

**Background:**

In Ethiopia, 17.6% of the population has disabilities. Research has largely overlooked students with disabilities (SWDs), focusing instead on caregivers, teachers and officials.

**Objectives:**

The current study sought to include SWD voices by examining their experiences and identifying barriers and enablers to education in Ethiopia.

**Method:**

A participatory action research approach with a critical theory lens, engaging SWDs in two phases was carried out. In phase one, student researchers received training through collaboratively developed modules on research concepts, questions, tools and data analysis. Student researchers identified, co-created, refined and finalised the study methods. In phase two, student researchers conducted 18 interviews and two focus group discussions.

**Results:**

Results indicated that physical, sensory and financial inaccessibility negatively influenced the social lives of SWDs, reinforced stigmatising societal perspectives and disadvantaged them compared to their peers without disabilities. Stigma led to feelings of hopelessness and social exclusion. However, having a supportive (‘threshold’) person or organisation, individual grit, love and trust in education and a desire to defy critics (i.e. positive revenge) contributed to student success.

**Conclusion:**

Early interventions are vital for supporting SWDs in engaging with education. A dedicated advocate can significantly impact their success.

**Contribution:**

This study contributes to disability studies and inclusive policy by centring the voices of SWDs in Ethiopia, highlighting their lived experiences. It offers practical insights into how systemic stigma, inadequate infrastructure and limited psychosocial support hinder inclusion, while support systems, personal resilience and early interventions enhance educational success.

## Introduction

Worldwide, people with disabilities (PwDs) have less access to education, employment and healthcare (World Bank 2023; World Health Organization 2022), but the dearth of inclusion of PwDs is most pronounced in developing countries (Wodon & Alasuutari 2018; World Bank Group 2018). It is estimated that 80% of PwDs live in developing countries (World Bank Group 2024). In Ethiopia, 17.6% of the total population – about 15 million people – live with disabilities (World Health Organization & World Bank 2011), and 95% of PwDs are estimated to live around or below the poverty line (Ministry of Labour and Social Affairs 2012).

While the Ethiopian state has declared its commitment to improving access to education over the past two decades (Ministry of Education 2012, 2016), only 3% of Ethiopia’s estimated 2.4–4.8 million children with disabilities (CWDs) go to school. Barriers include stigma among parents and educators, inaccessibility, rigid teaching practices, poorly trained teachers and the lack of adapted learning resources (Sida 2014). The work done to understand barriers has primarily centred around administrators, teachers and parents (Franck & Joshi 2016; Ministry of Education 2012, 2016) without consultations with students with disabilities (SWDs) to support and guide educational access efforts.

A recent systematic review (Egan et al. 2022) investigated the lived experiences of SWDs from low-income and middle-income African countries (LMIAC). Across these countries, out of 1821, only 10 studies that included first-person perspectives from SWDs were found. In many cases, a lack of resources created a barrier to inclusive learning, but SWDs attributed a lack of resources to multiple causes, including instructor apathy, a lack of instructor training and/or knowledge or a lack of institutional support. This review demonstrated the value of building support programmes on a foundation that includes SWDs’ articulated needs. However, this review was unable to uncover the lived realities that contextualise how the timing and types of support can amplify support.

Several studies conducted in Ethiopia across primary to secondary schools revealed that SWDs face inadequate assistive technologies, accessible infrastructure, specialised instructional materials and trained professionals (Esayas 2023; Simachew & Alemayehu 2024; Tadesse 2019). In higher education, SWDs also encounter negative attitudes, lack of facilities and materials, unsatisfactory exam accommodations, non-accommodative teaching methodologies and lack of skilled human power (Moges 2015; Tirussew et al. 2014; Yohannes 2015). The educational challenges of SWDs of the country are attributed to several factors such as: (1) lack of effective and proper policy implementation as policies directed at national level to implement inclusivity does not go to the ground when it cascades into different regions of the country because of disparities in resource and political commitment (Teferra et al. 2018); (2) problems related to governance as the country’s socio-political landscape is characterised by a complex interplay of ethnic diversity and historical governance structures (Addis 2025); (3) conflict and political instability as a result of prolonged conflicts that contribute to the increased the number of PWDs and insufficient support to SWDs (Assefa & Adamu 2024); (4) cultural constraints of disability as there is a perpetual belief that disability is the punishment of God for sins and/or bad dead committed by parents and or in their circles, consequently leads to restricted participation and stigma in education (Adugna, Ghahari & Lysaght 2025; Tirussew 2005); and (5) lack of advocacy and SWDs voice in which education has been offered to SWDs without engaging them as active agents for their education (Tadesse et al. 2020). At the University of Gondar (UoG), there are two categories of SWDs who are fully sponsored by the Mastercard Foundation (MCF) Scholars Programme and those who are supported by the Ethiopian government schemes. Thus, the university catered to both sponsored and non-sponsored SWDs. Those sponsored scholars have several benefit packages, including stipends, research funds, personal assistants and additional assistive devices (e.g. laptops) in addition to the services provided to all SWDs by the university. As a result, those sponsored and non-sponsored SWDs have quite different scenarios as funding is assumed to have significant impacts and thus should be studied.

This study is aimed at providing SWDs the opportunity to collaborate, relate, report and advocate based on their personal experiences from their educational journey in Ethiopia. The specific research questions guiding this study were: (1) *What are the retrospective, contemporary and prospective barriers and enablers to accessing education (at the primary, secondary and post-secondary levels) as related by SWDs completing a degree at the University of Gondar?* and (2) *How do the lived experiences of university education differ between SWDs who receive supportive funding from an international organisation and those who do not?*

## Research methods and design

We adopted a critical lens (Horkheimer 1972) within a participatory action research (PAR) methodology to explore the barriers and facilitators experienced by SWDs (Macdonald 2012). Students with disabilities were engaged as peer researchers in the research process in developing questions and interview tools and their application in data collection, where their agency and active participation were maintained. The student researchers (SRs) were engaged and empowered to be active partners (their own voices were heard), not passive participants throughout the research process. They were encouraged to analyse their own lived experiences, helping them to understand the relationship between circumstances, action and consequences in their own lives (Baum, MacDougall & Smith 2006; McTaggart 1997; eds. Reason & Bradbury 2008).

### Theoretical foundation

**FIGURE 1:**
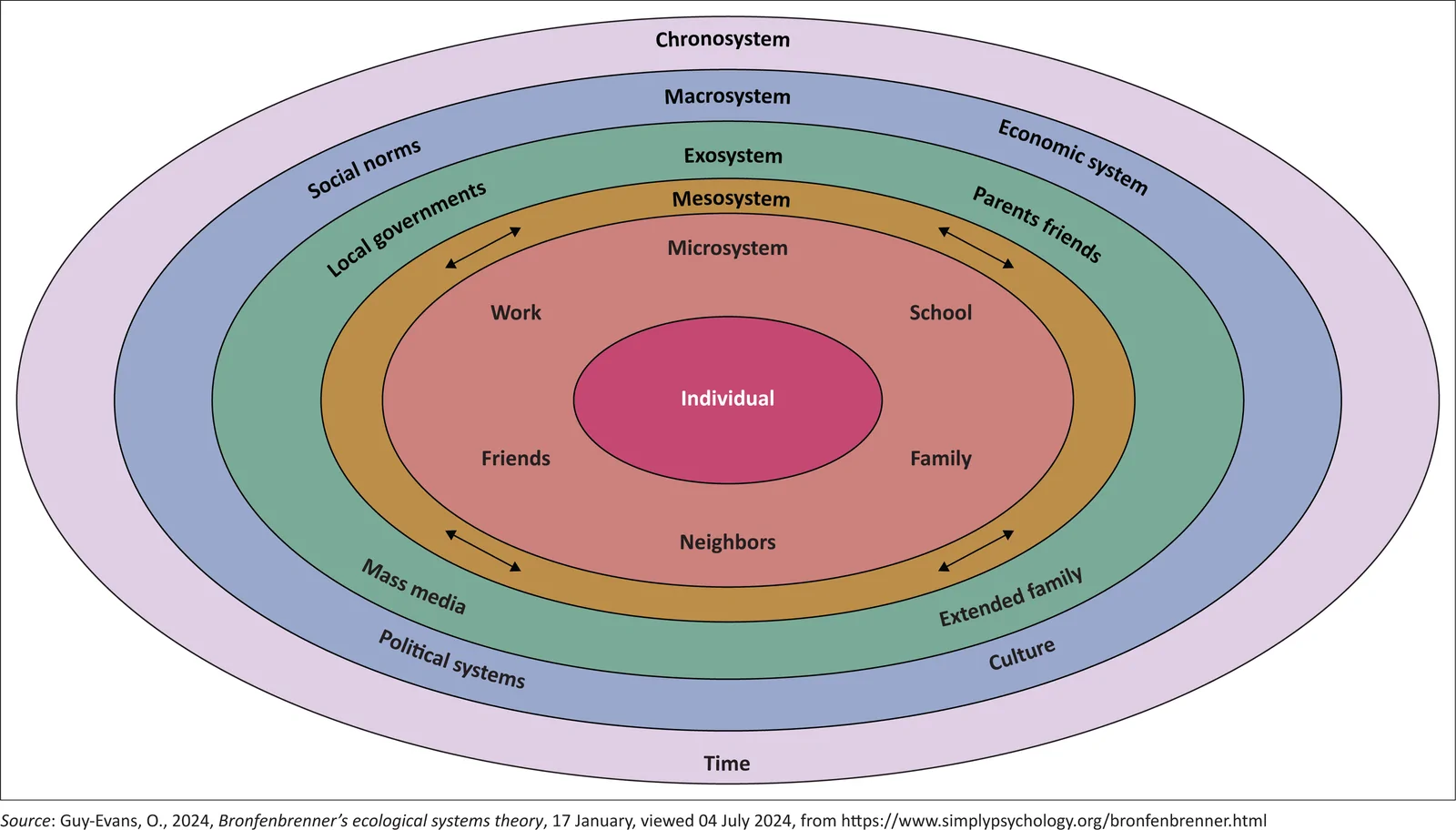
Bronfenbrenner’s bioecological model of human development (Guy-Evans 2024).

Informed by a critical paradigm and PAR approach, we adopted Bronfenbrenner and Morris’ Bioecological Model (Figure 1) (Bronfenbrenner & Morris 2006; Taylor & Ali 2017) to guide and structure this study. The ongoing and systemic discrimination of SWDs is something that must be understood across many levels, including SWDs’ perception of self (e.g. self-concept; i.e. *Individual level*), immediate connections (e.g. home supports; i.e. *Microsystem* level), (lack of) societal and social supports (e.g. societal pressures, peer relationships, mentors and bullying, stigma; i.e. *Mesosystem* level) and across the pervasive ableist culture (i.e. *Macrosystem* level). In applying the Bronfenbrenner model to our research, we found that the collectivist nature of Ethiopian culture necessitates placing parents’ friends and extended family within the *Microsystem* level, rather than the *Exosystem* level. In Ethiopia, parenting is not left to parents only, but it is a collective task given to extended family and friends. They all engage in child-rearing business with almost with equal engagement in nutrition, safety, security, early learning and care giving (Abesha 2012; Aemro 2015; Tafere & Chuta 2016; Yeshanew 2024). The experiences of SRs must also be set in the context of cumulative discrimination and ableism effects over their lifetime (*Chronosystem* levels).

### Study design

In this study, we adopted a collaborative, *in situ*, and exploratory approach of PAR research principles and methods. By avoiding prescriptive methods for data collection and analysis, we were able to guard against an ableist orientation that could easily become pervasive, condescending and disempowering.

In *Phase I* of this study, two modules were collaboratively developed and implemented with the six SRs and research team, each on average lasting 4 h. These modules provided foundational knowledge, the opportunity to discuss the value of a collaborative approach, normalise power equality and establish trust for all members of our group.

Student researchers conducted interviews and focus groups with other SRs and SWDs. Initially, SRs were invited to write reflections on their lived experiences, which informed the development of interview questions in conjunction with insights from *Phase I*. Subsequently, the SRs conducted a total of 18 interviews, including four interviews with fellow SRs, 12 interviews with non-SR SWDs and two focus groups (seven scholars and seven non-scholars) co-facilitated by SRs and faculty researchers with non-SR SWDs who were both Mastercard Foundation Scholars Programme scholars and non-scholars.

### Participant recruitment and data collection

Our approach encouraged the active data collection participation of SRs and faculty, aiming to bolster the transparency of our findings through continuous reflexivity (Dodgson 2019) and memoing (Razaghi, Abdolrahimi & Salsali 2015; eds. Reason & Bradbury 2008) by all stakeholders, including SRs, SWDs and faculty. Student researchers from the UoG were engaged without imposing any exclusion criteria related to the duration of SWDs’ studies or the nature of their disability. We used a sample stratification to ensure the inclusion of a diverse sample of SWDs on the research team. Additionally, a sampling grid was developed, considering various factors such as disability type, gender, academic year and discipline to guide the recruitment of SRs as indicated in Table 1.

**TABLE 1:** Demographic characteristics of student researchers.

Number	SRs	Sex	Disability type	Department	Year of study
1	SR1	M	Physical disability	Law	5
2	SR2	F	Blind	Social Work	4
3	SR3	F	Low vision	Law	5
4	SR4	F	Low vision	Sociology	4
5	SR5	M	Blind	Adult and Community Development	4
6	SR6	M	Physical disability	Pharmacy	5

SR, student researcher; F, female; M, male.

The recruitment of participants was carried out using multiple methods, including SWDs’ telegram groups, notice board postings and snowball sampling techniques or students’ social networks. The research team, including the SRs, provided prospective participants with detailed information about the study and consent forms in accessible formats, such as audio and written English and Amharic, and braille to ensure a thorough understanding among the SRs. Although English is the medium of instruction at the university, the data collection was conducted in Amharic, which is the first language of the participants. Thus, interpreters for Amharic and sign language were provided in interviews and focus groups to accommodate the linguistic diversity of the participants. Moreover, we made all necessary accommodations, such as Internet data to us Job Access with Speech (JAWS) software and assistants, to facilitate participation, implementing these adjustments wherever feasible.

During the development and implementation of modules, qualitative data were collected using audio and video recordings through Zoom® meetings between researchers and SRs. Additionally, comprehensive meeting notes were taken and subsequently validated by all researchers, providing further context and insights into the meeting. These interactions were grounded in the principles of dialogue, fostering informal and open conversations to build effective working relationships among the research team and SRs (Wosk 2019; Yankelovich 2001). Upon completion of the modules, the recorded meetings and feedback from SRs during the modules were analysed and discussed between the team.

### Data analysis

Drawing on the principles of Constructivist Grounded Theory (Charmaz 2006; Denzin & Lincoln 2018), we engaged in an iterative process of transcription, translation (Gawlewicz 2019), thematic analysis (Braun & Clarke 2021) and axial coding (Corbin & Strauss 2015) throughout both phases of this study. All interviews and focus groups were transcribed in Amharic and translated into English by professionals from UoG. All of the Amharic transcribed and translated data were checked and validated by SRs.

Dialogues and reflective sessions with SRs and the research team during the module creation and implementation were instrumental in formulating the interview and focus group queries for the subsequent phase. Following the completion of the two modules, meeting recordings, notes and reflexivity documents were reviewed by the research team, allowing us to narrow and refine our research scope. During the second module, member checking and comprehensive discussions were used to iteratively develop and re-develop the protocols and observation tools. The evolving research questions, tools and methodologies were consistently framed within the Bioecological Model of Bronfenbrenner and Morris (2006).

Through constructivist and critical perspectives, we adopted a cyclical process of data analysis, coupled with the ongoing reflexivity among PAR members, which allowed for a multifaceted exploration of subjective realities at the intersection of various biases. Initally, an analysis manual tailored for the PAR team was developed by a research member with knowledge of PAR methodologies. Ethnographic (Rivoal & Salazar 2013) and informal data, including field notes, reflexivity journals, audio recordings, drawings and photo journals, were collectively contextualised and interpreted within our analysis approach. Our primary analytical method was thematic analysis (Braun & Clarke 2021), complemented by axial coding, inter-rater reliability and reflexivity approaches (Corbin & Strauss 2015; Denzin & Lincoln 2018; Glaser & Strauss 2017; Miles & Huberman 1994).

Six research team members engaged in a collaborative coding process over 5 days to complete preliminary coding and establish an initial codebook. This process involved rigorous discussions with the PAR team and SRs to refine the codebook, ensuring it accurately reflected the experiences and perspectives of SWDs. Each data transcript was coded in full by two research team members, leading to the formation of preliminary codes and categories. Then, the six research team members collaboratively developed the primary themes from the data, with constant input from SRs to validate the analysis and findings. Students with disabilities within our team were provided structured opportunities to critically interpret codes, categories and themes, ensuring the conclusions drawn were reflective of the participants’ realities. NVivo 14 software (Lumivero 2023) was utilised for coding, with inter-rater reliability achieved through the independent coding of interviews by pairs of researchers (Braun & Clarke 2006; Miles & Huberman 1994). Discrepancies in coding between researchers were resolved through discussion until a consensus was reached, ensuring a robust codebook was developed.

Using thematic analysis facilitated the comparison, contrast and synthesis of codes and/or categories across the intersected experiences of our participants, revealing overarching principles and forces conducive to addressing through significant funding avenues like the Mastercard Foundation. Themes derived from critical thematic analysis, grounded in Bronfenbrenner’s Theory, laid the groundwork for our initial findings. Finally, the data were then further analysed against the educational trajectories of SWDs (i.e. elementary and/or boarding, middle, high and post-secondary education phases). This chronologically situated analysis facilitated integrating and juxtaposing emergent themes and categories.

### Ethical considerations

Ethical clearance to conduct this study was obtained from the University of Gondar Institutional Review Board (IRB) (No. V/P/RCS/05/2743/2021).

## Results

The narratives, including both SRs and non-SR participants, showcase various familial, peers, classmates, community, institutional, instructional and interpersonal relationships across all education levels.

A lack of accessibility was described as a pervasive and omnipresent influence across every aspect of their lives. Provided a clear message that inaccessibility is unacceptable and advocacy for accessibility equity is essential. The lack of accessibility negatively influenced their social lives, supported stigmatising societal perspectives and put them at a severe disadvantage relative to their peers. This resulted in feelings of frustration, anger and despair, yet unfair disadvantages were also seen as surmountable and have been used as an opportunity to advocate for positive change. Participants took actions to overcome accessibility challenges and were simultaneously inspiring and heartbreaking. The three main categories of inaccessibility were physical, sensory and financial.

Participants consistently reported that there were pervasive and dehumanising societal stigma and abuse. Alongside low expectations and the tendency to seclude SWDs from society by family and community, this stigma and abuse contributed to lowered confidence, self-stigmatisation and ultimately the erosion of self-concepts, including the idea of who they are physically, emotionally and socially as meaningful and productive members of society. Across most journeys, participants identified a specific and pivotal event where individuals provided emancipation from societal oppression and were essential to facilitating the transition from self-concept erosion to the development of an accurate self-concept as productive and capable members of society. These events and individuals supported participants as they crossed the symbolic threshold to becoming scholars and, as such, are referred to in this paper as *Threshold Facilitators*. Ongoing educational success initiated a cycle of self-concept development through educational goal setting, increased self-efficacy and achievement. Through educational transformation, participants reported that they were able to view societal ignorance and fear as an opportunity and platform for change.

### Physical inaccessibility

Findings from SRs and participants have more or less similar lived experiences in the trajectory of primary school to higher education. It is, therefore, possible to say that the findings from these two participants were found to be convergent. Participants painted a compelling and concerning picture of natural and built inaccessibility of schools. Without mobility aids, students were required to navigate hilly and muddy landscapes by ambulating with their hands. As one participant reported:

‘I moved places on my hands and knees. My hands got dirty when I walked on them. I often wore a scarf and wiped off the dirt with it.’ (SR1)

The lack of accessibility within the natural environment also impacted school attendance and learning and perpetuated stigmatising attitudes. A participant noted how a lack of accessibility was humiliating, physically harming and made it challenging to attend school:

‘In primary school, the place I used to live was a rural area with up and down topography. Although I went to school, I used to use both my hands and feet. My friends used to avoid stones to prevent obstacles. The place was stony and full of thorns.’ (P1)

On other occasions, students did not have peer (or other) support and were often left in perilous positions. The physical and educational inaccessibility challenges of the natural environment were exacerbated in communities where:

‘[*P*]eople used to say [*things such as*], [*i*]s this girl a human [*abled*], can she be successful in education and be an independent one?’ (P2)

Built structures, including classroom learning environments, were also challenging for participants, especially once they matriculated into university. There were different experiences in elementary school; some students found the single-level buildings were more accessible as they did not have:

‘[*U*]pper flats [*they were*] comfortable though [*I*] was walking on my hands.’ (P2)

### Inaccessibility to inclusive and/or assistive technology

Inaccessibility was specifically challenging for those with visual and auditory impairments as recorders, electronic readers, braille, hearing-aid devices and sign language interpretation were often unavailable. The lack of tools required was compensated by the support of friends, as indicated by one participant who indicated:

‘[*I could not*] reach this level [*of education*] without the support of my friends.’ (P6)

Moreover, participants needed to cultivate friendships just so they could access the information needed for their studies. One participant describes this process in detail:

‘There were not any friends with whom I could study, help me (dictate me; read for me), and there were not any access including books. However, whether there is access or not, more important thing is you must use your technique as well as your tactic to adapt yourself with the challenges. For example, you need to be sociable with the students just in order to get their support, so I became sociable and able to join many non-blind students instead of being close friend with students with visual impairment students.’ (P5)

Although strategic approaches such as this are laudable and demonstrate creativity, they are also disempowering and make studying more of a challenge. One participant succinctly sums up the challenge of tool accessibility in elementary and high school by noting:

‘Except in university, voice recorder and computer are unavailable at lower classes.’ (P9)

This sentiment, with some minor exceptions, was ubiquitously described by all participants. Frustratingly, even when tools were available, stigmatisation and/or ignorance resulted in the lack of availability of these tools:

‘Principals and teachers. There would be 20 or 30 or 40 computers in the school, out of which they could have made 2 or 3 computers accessible to SWDs. However, there was no such good will and positive idea. They may not know Jaws software; besides they did not have a thought of we can learn and get something by touching computers. At least they should have shown us keyboards.’ (P4)

As stated by a participant, part of the problem may have been that teachers lack knowledge. In one participant’s experience:

‘[*T*]here was not a single professional and the teachers did know what brail was and neither did I. I never knew whether brail is useful or harmful. Neither did anybody tell me, nor did I ask about it.’ (P1)

As a result, when students matriculated to university, where more tools were available, many of the students were unable to productively use these tools and as such were required to learn technological skills while completing schoolwork.

### Financial exclusion

A key challenge in elementary and high school was:

‘[*I*]nsufficient financial resources to cover house rent and food expenditures.’ (P8)

The heroic, strategic and heart-breaking activities participants used to cope with poverty included selling gum, lottery tickets and soft drinks, begging, eating food discarded from restaurants, shining shoes, sleeping in cemeteries and continuing habitation with abusive family members. One participant summed up the compounding effects of financial constraint on their quality of life and quality of education as follows:

‘As I told you that money (350 birr [*provided by the government*]) was not enough. There was hunger. Since I was hungry, I was missing classes to go to rural area to ask [*for*] food, [*and that could not be scheduled*]. I was missing classes, and it was difficult to cover the missed class. There [*wasn’t anybody*] to take note[*s*] and record as there were no recorders [*at*] that time. It was difficult to get readers, leave alone to write things in Brail. Thus, it was very challenging to move on by overcoming all these challenges.’ (P4)

During these trying times, many young students:

‘[*W*]hom I knew in lower classes [*became*] street individuals due to acute economic problems and being refused school registration for lateness.’ (P7)

### Stigma within the microsystem

Families and communities believe that church education is the only ideal place for SWDs. In some cases:

‘[*The attitudes of their*] family, friends and members of [*the*] community [*made them*] feel different.’ (P12)

Parents widely held the belief that:

‘[*PAR-SRs would*] will be exposed to further dangers if they go to regular education [*and that they should be kept out of sight from educational and social communities, as*] they didn’t want other people to see [*them*]’. (P2)

It should be stressed here, in addition to facing stigma and abuse within their families, community members also ostracised both the families and the participants themselves. One participant stated:

‘There was and is a thought in the community that PwDs should not be in the education [*system*] and [*rather they should*] assume employment around churches and religious institutions.’ (P8)

This participant recalls his father saying:

‘Why [*do*] you want to go to school? If you want to [*learn*], I recommend you [*enrol in*] church education to [*become a*] church scholar. And if you don’t want to [*do this*], I will [*not support you*].’ (P8)

On the rare occasions that family members did attempt to support their children, the community would react negatively. Alongside the belief that SWD were unable to learn and engage in society, members of the community and family also believed that disability resulted from sin. One participant recalled that after a childhood scuffle, a peer’s father insinuated that his disability was because of his dangerous character, saying:

‘God took the legs of the snake knowing his heart.’ (P6)

### Stigma at the exosystem

Teachers in elementary and high school, as described later, were often key to supporting participants’ transition into positively reinforcing cycles of self-concept development. However, there were also many occasions when teachers stigmatised SWDs through overt abuse and low expectations. A participant related a principal who refused to allow them to sit at the front of the classroom, but instead said that:

‘The chairs are compacted and there is not enough space to freely pass, so let your friends drag you to the back chairs.’ (P6)

One participant recalled a time where the teacher’s abuse took the form of enabling the stigmatisation and ostracisation of others. Teachers also stigmatised students through low expectations and accusing:

‘SWDs [*of*] com[*ing*] [to class] having others do their assignments, not by themselves.’ (P12)

One participant noted that in this regard:

‘[*I found*] teachers in the lower classes [*were*] better.’ (P4)

Although potentially less detrimental at the university level because of participants’ profound strength and their accurate self-concept as capable students, SR provided a salient example of how this continued stigmatisation is both unfair and frustrating:

‘Last time, my mother was sick, I missed one midterm exam as a result of paying her a visit. When I returned, I went to the teacher’s office and while I was telling him my case he saw my [*disability*]. Immediately, he said, “You guys are always busy at disturbing/begging others!”… The teachers see everything [that we do] as a burden to them, and think we are being favored in grades. It is disgusting. I am not inferior to my classmates. Everyone gets grades based on his/her effort, so do I.’ (SR6)

In contrast, educational support and the lack of stigma within the boarding school system were clearly identified. Although these schools provided less opportunity to develop relationships with students without disabilities, they were fundamental to student skill development and the development of an accurately positive self-concept as a capable person and student. One participant nicely summarised this sentiment:

‘I attended my school from grade one to grade six at boarding school. During my stay there, things which contributed to change my personality development were the people who manage me, the books which we were reading, things we were observing, the different games we were playing, and others. All these had meaningful contributions to my personal development, that is, all these made me become strong enough to overcome challenges facing me [*at*] this time.’ (P10)

### Stigma at the macrosystem

As such, there was limited discussion of stigma generally within the Ethiopian culture, and as such, there was little evidence that participants’ direct interactions with macrosystem stigma (e.g. news and media) directly impacted their educational journey. However, participants acknowledged the importance of systemic bias at the macrosystem level:

‘[*T*]he first problem is the society itself. There is an attitude that SWDs are unable to do anything.’ (P9)

Male and female participants indicated that sexism and sexual abuse were an additional challenge for females. One participant was:

‘“[*N*]ot willing to tell [*you*] the place or other details” but had “several challenges including sexual harassment…[*if she*] had not been strong [*she*] could have an illegitimate child and dropped out from education.” (Participant number obscured for confidentiality)

A male participant also noted that:

‘[*F*]or female SWDs it was difficult because they were not going outside home lest they be exposed to further adversity.’ (P8)

This is a particular problem once they leave their family home as:

‘[*U*]nless you are strong, leave alone with a disability, it is very difficult for those females [*without disabilities*].’ (P1)

### Self-concept erosion

The outcome of stigma and abuse, especially prior to and during early education, was that participants developed a self-concept of being inferior and unable to interact with peers, unable to learn and ashamed of their disability. The self-concept erosion starts from a negative reaction of parents, relatives and neighbours to a CWD, which leads to rejection and negative emotional and psychological feelings. Self-stigmatisation was a problem as they:

‘[*S*]tarted to believe [*others*] when they told [*them*] that [*they*] would be unsuccessful through education.’ (P2)

Collaborators had a general feeling of lacking, and as one participant noted:

‘[*A*]ssociated all of [*their*] failure[*s*] with being a person with a disability.’ (P12)

The isolation and hopelessness that participants felt were effectively summarised by one participant who said:

‘When I was at primary school, first to sixth grade, I did not have a firm belief and hope. Therefore, I was fighting with my challenges only by myself.’ (P6)

### Threshold facilitators

Threshold facilitators are individuals who provide supportive guidance and play key roles in helping SWDs build resilience throughout their journey, enabling them to reach their current stage. Participants reported a broad range of threshold facilitators that allowed them to start or continue their studies. Most members of their community and/or family (with notable exceptions) offered minimal protection against pervasive social stigma and abuse.

### Entering education

On two occasions, family members supported participants by rejecting familial pressures and enrolling them in primary education. In one case, the participant’s aunt travelled with them to the capital city of Addis Ababa and enrolled her in a specialised boarding school. On another occasion, the participant noted that they:

‘[*H*]ad a sister and it was her who carried me when I fled my home.’ (P12)

This participant was forced to flee persistent community and familial abuse, concealment and pressure to attend church school.

#### Coping with challenges of elementary and high school

Threshold facilitators were crucial during elementary and high school. For three participants, parents provided support during high school. For one, their mother sold soft drinks to pay for their schooling; the second participant’s mother:

‘[*U*]sed to tell me as if nothing has happened and my disability never prevents me from doing anything.’ (P11)

The third participant’s mother notes that:

‘[*F*]ather used to bring [*me*] firewood [*via*] his donkey after I joined high school for about four years including one extra year because of COVID-19 pandemic.’ (P1)

Teachers also provided key supports well beyond their professional duties:

‘I had teachers who were like family that used to support me a lot. I am here due to their efforts. After grade 7, I attended grade 8 at a district school renting a house. The house rent was 30 Birr, and my teachers were used to pay it.’ (P10)

Other acts of key teacher support included taking a student to Addis Ababa for treatment, providing moral support and encouragement and teachers with disabilities providing lived experience and strategies. Overall, 5 of the 12 participants in this study attributed their continuance in education to the support of their teachers.

#### Self-concept development

As previously evidenced, the key reason why participants matriculated to the university level is their personal strength, strategic planning for entering education and resilience. Unanimously, students credited their evolving self-concept as capable, strong and inspiring members of society to their educational success. Education created a positively reinforcing cycle that was enhanced by starting school on time alongside peers, and prior to self-stigmatisation, unfortunately:

‘[*I*]f we start [*school*] old, we might be consumed by several [*negative*] thoughts.’ (P2)

Throughout elementary and high school, participants reiterated the importance of their educational experiences. This was summed up nicely by one participant who stated:

‘Before I started education, I was thinking a PwD is unable to do [*anything*] and he/she will be dependent on others for a living. But, after I started learning I have witnessed lots of changes. I have developed awareness that a PwD can live an independent and working life as any other person without disability.’ (P9)

Participants attributed their overall success to education, as bluntly summarised by one participant who noted:

‘My hope, the first and ever is my education. If I had not been determined in my education, I could have been on the street. As you see me, I am here.’ (P1)

Education also developed resilience and the ability to disregard societal ignorance. A participant noted:

‘I think education and the [*school*] community have changed me a lot. At first, [*people will be*] scared of the challenge, [*b*]ut, soon you will get adapted to it and you start to ignore it.’ (P11)

### Positive revenge

All participants indicated the importance of revenging the abuse they received throughout their lives by demonstrating their effectiveness. The juxtaposition of the words positive and revenge connotates the motivational benefits of participants demonstrating their success. The term was coined by SR, who stated:

‘There are still challenges, yet I think only about taking positive revenge on those who doubted my success. I always had the courage to do what I believed not what people wanted me to do.’ (SR3)

Positive revenge was demonstrated in many ways. One SR explained the joy he felt in providing financial support to a step-father who had abused him in their childhood by stating:

‘Thanks to God, when I send money through bank to my mother [*who was very supportive*] I intentionally call her through the mobile of my Stepfather. It gives me untold happiness!’ (SR6)

One participant, whose father was berated by the community for sending him to (public) education, relates the joy of demonstrating:

‘[*S*]uccess to those people who used to say to my father that he killed and spoiled me the fact that he enrolled me in school; not in church education. Currently, those people who used to say you are killing your child, started to love me. They say, [*they are on their*] way! Go to school [*supporting children’s enrolment to school*].’ (P11)

Finally, participants demonstrated the clear connection between positive revenge and the development of self-concept by indicating:

‘To me the meaning of self-efficacy is, being able to exhibit yourself as self-reliant [*person*], becoming what you want to be, demonstrating your achievement, and showing the community that does not believe in our potential.’ (P1)

### The power of university

Matriculating at university was seen by participants as an important milestone, which motivated their progress through lower classes. The motivational importance of university was summarised by one participant who said:

‘[*A*]fter I have joined university, I have a belief that my dream will come true.’ (P3)

Similarly, another participant demonstrated the importance of university, especially at the end of high school, by indicating:

‘[*S*]tarting from grade 11 to 12, I started to get chance to set my dreams and goals. I was dreaming to join a university and study Law. Thanks to God my dream has come true!.’ (P5)

University matriculation also played an important role in positive revenge motivation. One participant summarised this sentiment by noting:

‘From university when we go to our hometown for summer vacation and break, [*our communities have*] positive attitude[*s*]. They now believe we can learn and be somebody. It does not mean however, there are no influences which are not solved yet, but we have to appreciate the change.’ (P4)

### Current opportunities and challenges at the University of Gondar

The focus group discussion was made entirely to get an answer for the research question 2. That is why findings from the analysis of the focus group discussion were not mixed with the in-depth interview analysis presented in the preceding sections. The focus groups that included participants with and without Mastercard Foundation funding highlighted key learning challenges and facilitators at the UoG.

#### Financial and sensory accessibility

A clear and persistent finding was that funded students were well funded (to the extent that they were able to send money home) and received the accessibility tools they required. A participant in the MCF-funded Focus Group (FG) noted:

‘[*B*]efore, we join UoG, service provision and after joining UoG, service provision is not comparable.’ (Participant number obscured for confidentiality)

Another participant from the same FG noted that:

‘[*A*]fter I joined the university, as my friends said, there is no financial problem.’ (Participant number obscured for confidentiality)

Unfortunately, there was a distinct feeling of inequity in support and a feeling that there were two classes of students. One participant from the unfunded FG indicated:

‘Besides, when skill training is given for scholars, we must be included in the trainings. We got money in different ways, but we do not get lessons that change our awareness and broaden our thinking. We do not get training as much as we want.’ (Participant number obscured for confidentiality)

Similarly, MCF-unfunded FG participants noted accessibility inconsistency. In particular, the availability of braille was noted to be a particular challenge for MCF-unfunded FG participants. In contrast, participants noted that there were residual benefits that they could access. One MCF-unfunded collaborator noted:

‘In this university, there is Mastercard foundation which pays for readers and related issues, so we share these benefits though we are not scholars. For example, when tutorial is arranged for scholar students nobody prevents us from attending all tutorial classes.’ (Participant number obscured for confidentiality)

#### Physical accessibility

There was a general concern about the lack of physical accessibility of the UoG campuses, although it was also noted by one MFC-unfunded participant that:

‘[*I*]t [*physical accessibility*] is better at the UoG as compared to other universities in Ethiopia.’ (Participant number obscured for confidentiality)

In addition it was noted by one MFC-funded participant that there was a feeling that the accessibility of dormitories was better as:

‘PwDs can make a group of six and pick whatever dorm they want.’ (Participant number obscured for confidentiality)

A key accessibility challenge was the lack of elevators to classrooms, offices and libraries. An MCF-funded collaborator summarised this impact by noting:

‘[*T*]he library has no lift. Now the buildings are tall but have no lift. Whether it is classroom or office it is the same. When we want to visit offices at the last floor, we cannot because the buildings have no lift. Thus, when buildings are constructed, they are not convenient for SWDs.’ (Participant number obscured for confidentiality)

Roads were not challenging in and of themselves, and in fact, the MCF-funded FG participants noted:

‘We were surprised when we joined the UoG because the campus is great, and its roads are good and comfortable for PwDs.’ (Participant number obscured for confidentiality)

The challenge with roads was primarily drivers and parking practices; one MCF-unfunded FG collaborator indicated:

‘In term of cars still the problem is the same as lower classes. They park everywhere. No matter what we discussed with top management over the car parking issue it is not resolved yet. The infrastructure for the parking is underway, only God knows when it will be completed. Though there are good drivers there are however some who drive inside campus carelessly as if they are driving in the public asphalt road. Last year one of our friends got car accident.’ (Participant number obscured for confidentiality)

MCF-funded FG participant indicated that students with hearing impairments were restricted to a single programme, which is Special Needs and Inclusive Education, and as such were unable to study areas aligned to their diverse interests.

#### Psychosocial support

There was a consensus between MCF-funded and unfunded FG participants that there was a lack of support from many instructors at the UoG. A participant from the MCF-funded FG simply summarised the feeling of students by saying:

‘[*Here*] such individual support is almost null [*and that*] before the university in lower grades, it is better in terms of moral support.’ (Participant number obscured for confidentiality)

An MCF-unfunded participant explained how this problem impacts their studies:

‘The lecturers, please forgive me for my statement! They are unfriendly. They usually punish by reducing marks. As you know in university a reduction of one mark has significant negative impact.’ (Participant number obscured for confidentiality)

Alongside a general lack of support for students, collaborators in the unfunded MCF FG noted that a culture of university staff discrimination has been created between funded and unfunded MCF students. One participant identified that:

‘The problem is when we go to different offices they ask us, “Are you Mastercard Scholars?” when we say, “no,” some of them give us an ugly face. I do not know what kind of response they would give us if we were [*MCF-funded students*]? And some of them say, “is it not difficult for you to compete with Mastercard scholars?” I don’t know what special thing is there with Mastercard scholars?! They make us question ourselves that way.’ (Participant number obscured for confidentiality)

In contrast to experiences with instructors within the UoG, funded and unfunded MCF students have developed a supportive community that transcends funding status. In fact, wealth sharing and mutual support have become a standard practice between all students. One MFC-funded participant describes the beautiful culture that has been developed between groups as follows:

‘There was one female SWD who lost her money, and she was totally saddened. Instantly, two Mastercard scholars out of their pocket handed over the same money to the girl and they made her relieved of her sad mood immediately. This came from nowhere but from our strength, strong relationship, and empathy with one another. It is impossible to leave the girl saying I don’t care. The other is, scholar SWDs bought a laptop for two Law SWDs that the students could not do their studies only by recorder [*as the discipline is Law*]. This is something. A big thing beyond measure! This is our friendship at its best. Me for example, I do not record the class properly. So, I borrow from my classmates.’ (Participant number obscured for confidentiality)

Accessibility challenges are also met with compassion, love and cooperation. For example, an MCF-funded FG participant related that:

‘I move in a wheelchair and a blind student pushes the steering wheel of my wheelchair at the back, I guide him with directions. I am very happy because when we help each other, it is a love, I love it.’ (Participant number obscured for confidentiality)

## Discussion

The nature of the challenges facing SWDs generally, and specifically in low- and middle-income countries, including those in Africa, is well established (Egan et al. 2022). To address these challenges, funders such as the MCF play a crucial role in supporting SWDs financially, socially and psychologically and through the provision of accessibility tools and assistive devices (MCF 2024). However, notwithstanding this work, there is an acknowledgement by the MCF that ‘research is needed to understand what types of interventions are needed to improve education and employment outcomes among PwDs’ (International Centre for Evidence in Disability, Addis Ababa University, and Mastercard Foundation 2023). The goal of our PAR research approach was to learn from SWDs at the UoG who have successfully traversed the perilous journey to university education. The power, respect and love shared between our team ensured that SWDs, staff and faculty equally contributed their strengths and insights. We believe these insights and stories are essential to guide funding agencies to provide the right resources, to the right people, at the right time.

### Early intervention

Participants in this study unanimously attributed their success to developing qualities of resilience, perseverance, and advocacy for themselves and others. When they viewed themselves as productive and inspiring members of society, they were able to overcome potentially devastating barriers. A key step to developing resilience was the support of threshold facilitators to begin and/or enable sustainable engagement in education (Ng et al. 2015). In turn, educational success was identified as an important factor in self-destigmatisation, and the development of self-concepts needed to thrive. There were many participant stories, and there is good statistical evidence (Jones, Seager & Yadete 2021), that SWDs who remain concealed, self-stigmatised, and ostracised by society live on the margins. Thus, our PAR participants advocate for early interventions that support SWDs to enrol in secular or regular education. It is important to fund threshold facilitators that can advocate for young SWDs, and demonstrate to communities, families and SWDs alike that PwDs can, have, and continue to be societal leader. University graduates with disabilities are excellent ambassadors (Kimball et al. 2016) and alongside minimal financial support, community engagement and individual mentorship threshold facilitators will be able to address stifling individual and microsystem (self) stigmatisation.

### The means of life

Although MCF-funded participants were grateful for generous financial support at the university, they noted that it was insufficient without reciprocal psychosocial support, which empowers SWDs and improves their accessibility to and belongingness in higher education (Akoto et al. 2022). Moreover, the students who now receive generous allotments first had to survive through periods of starvation and degrading strategies to acquire food and housing. Our PAR participants want to emphasise the importance of sufficient funding across all stages of education, including university. The financial support need only be sufficient to allow SWDs to concentrate on their studies instead of being required to spend time, energy and self-esteem acquiring the means of life.

### Accessibility

Tools for physical and sensory accessibility are essential for SWDs. Like any student, SWDs have preferences for which tools to use based on ways of learning, personal skills and educational contexts. There have been many debates in the literature between academics, educators and policy makers regarding how to balance accessibility with socialisation in educational environments and how best to support SWDs. Unfortunately, as identified in our team’s 2022 systematic review, there were no studies that collaborated with SWDs to explore their lived experiences to identify what strategies they needed (Egan et al. 2022). Our results clearly demonstrate that SWDs, especially in upper elementary and high school, are more than able to determine the tools and environments that they need. In fact, collaborators in our work were able to cultivate relationships, improvise accessibility tools and strategies and rationalise unthinkable hardship to meet their goals. Although traumatic, the silver lining expressed by collaborators was self-knowledge, autonomy and self-advocacy. Funders have the opportunity to provide a minimum set of accessibility tools and training, listen to the true experts, the SWDs themselves and, within the limits available, provide tools (even communal tools) that are requested.

### Infantilisation

Students with disabilities in Ethiopia are required to precociously develop emotional, cognitive, social and psychological resilience and independence. To navigate daily life, they must quickly learn how to challenge societal ignorance rooted in fear and be resilient against prejudice and misconceptions about disability. They are compelled to exceed the performance of their peers to persist in education and overcome the barriers of stigmatisation and inaccessibility. Our participants were frustrated by previous research projects where data were collected about them, and conclusions and recommendations were drawn without them. This resonates with the motto of the 2004 International Day of Persons with Disabilities, ‘Nothing About Us Without Us’ Governmental and institutional conversations about how to serve SWDs rarely engage collaboratively with those they mean to serve. In short, those for whom society demands precocious maturation for survival are continually infantilised by entities that purport to be serving their interests. The community of sharing and mutual support that has blossomed at the UoG provides a clear and present example of the organic strength of SWDs who are given the means to control decisions and organise themselves.

From the overall findings, it can be inferred that although the study was conducted at UoG, the SWDs come from all regions of Ethiopia. This diversity reflects a broader representation of the country within wider continental contexts. Therefore, the findings of the present study can serve as baseline data for further research in other low- and middle-income countries across the continent and beyond. In these contexts, governments and other key stakeholders should strengthen their commitments and take deliberate action to minimise, and, where possible, eliminate, existing power, agency and structural inequalities. Such efforts should begin at the pre-kindergarten level and extend through university education, as well as across all areas of life. These initiatives should actively engage and empower SWDs through a shared vision and common goals to achieve accessible and inclusive education, ultimately fostering inclusive societies and sustainable development across the continent and beyond.

## Conclusion

The complex challenges encountered by SWDs in low- and middle-income countries necessitate a comprehensive approach that extends beyond financial assistance. Our study underscores the significant role of early interventions and support systems in nurturing resilience, self-advocacy, social inclusion and academic achievement among SWDs. Findings from our participants at the UoG underscore the importance of sustained funding to assist SWDs throughout their educational journey, ensuring that they can concentrate on their studies without the worry of meeting basic survival needs. Customised accessibility tools that cater to individual preferences and local contexts are indispensable, and donors should directly engage with SWDs to comprehend their lived experiences and specific requirements based on SWDs’ types of disabilities. Additionally, it is vital to involve SWDs in research and policy development as active contributors and utilise their inherent strengths and insights. By providing appropriate resources at the appropriate time while honouring the autonomy and expertise of SWDs, we can establish more inclusive and transformative educational environments that enable these students to prosper and take the lead.
